# Viscous Rheological Behavior of Nanosuspensions of Fumed Silica Nanoparticles and Cellulose Nanocrystals

**DOI:** 10.3390/nano15191468

**Published:** 2025-09-25

**Authors:** Rajinder Pal, Hanie Alizadeh

**Affiliations:** Department of Chemical Engineering, University of Waterloo, Waterloo, ON N2L 3G1, Canada; halizadeh@uwaterloo.ca

**Keywords:** viscosity, rheology, non-Newtonian, suspension, nanosuspension, nanofluid, fumed silica, cellulose nanocrystals, nanoparticles, nanocrystalline cellulose

## Abstract

The viscous rheological behavior of suspensions of mixtures of fumed silica nanoparticles (N20) and rod-shaped cellulose nanocrystals (NCC) were studied experimentally. The fumed silica concentration varied from 2 to 11.3 wt% and the NCC concentration varied from 0.99 to 6.73 wt%. The suspensions of pure fumed silica, pure NCC, and mixtures of N20 and NCC were non-Newtonian shear-thinning in nature. The viscosity versus shear rate data of all suspensions of pure and mixed additives could be described satisfactorily by a power-law model. The consistency and flow behavior indices of the suspensions were strongly dependent on the concentrations of both N20 and NCC. While the consistency index increased sharply with the increases in additive (N20 and NCC) concentrations, the flow behavior index generally decreased with the increases in N20 and NCC concentrations. Thus, the suspensions became more shear-thinning with the increases in N20 and NCC concentrations. The shear-thinning of suspensions was due to two different mechanisms: the orientation of rod-shaped cellulose nanocrystals in the flow direction with the increase in shear rate and the break-up of large agglomerates of fumed silica aggregates with the increase in shear rate.

## 1. Introduction

In the formulation of many products of practical importance, thickeners or rheological modifiers are utilized due to one reason or another [1,2,3,4,5]. For example, in the food industry, development of products such as sauces, dressings, and dairy products requires firm control of texture and consistency. The products must have the right mouthfeel and stability during processing and storage. This can be achieved by manipulating and controlling the rheology of the product with the help of edible thickeners. Control and manipulation of rheology with the help of thickening additives also plays a vital role in the formulation of drugs, their processing, administration, and controlled release of active ingredients. Liquid thickening is also commonly used in the adjustment of diet for individuals suffering from dysphagia, which refers to difficulty in food swallowing. The thickening of liquid food prevents choking and keeps liquid food from entering their airways [6]. The control of the rheological properties of cosmetic products such as lotions, creams, and gels is essential to ensure that the products can spread easily and feel pleasant on the skin. In oil well drilling operations, thickeners are used to regulate and control the performance of the drilling fluid. In the formulation of products in the form of suspensions and emulsions, thickeners are often used to control the separation of phases and deterioration of products in a gravitational field. Heavy particles and droplets of suspensions and emulsions tend to settle or sediment, whereas light particles and droplets tend to rise or cream. By thickening the matrix liquid, the sedimentation or creaming of particles/droplets under the influence of gravity is reduced substantially in accordance with the well-known Stokes’ law [7].

According to some estimates, the market for rheological modifiers or thickening agents on an annual basis is more than 10 billion USD [5] worldwide. The commonly used rheology modifiers or thickeners are polymers, clays, and surfactants [3,4]. However, due to environmental issues and the general trend towards sustainable materials, nanomaterials (nanoparticles, nanocrystals) are coming up as a new class of cost-effective rheological modifiers and thickeners. The incorporation of nanoparticles and nanocrystals into liquids can alter the rheology of liquids substantially and provide the desired thickening effect.

In this work, we have utilized environmentally friendly and sustainable nanomaterials, fumed silica nanoparticles, and nanocrystalline cellulose as rheology modifiers for aqueous liquids. The viscous rheological behaviors of suspensions of fumed silica nanoparticles, nanocrystalline cellulose (NCC), and mixtures of fumed silica nanoparticles and NCC are investigated in detail. A broad range of additive (fumed silica and NCC) concentrations is covered. A good understanding of the viscous rheological behavior of suspensions of single and hybrid nanoparticles is important in the production, processing, and applications of nanosuspensions. It is our understanding that this is the first study to explore the viscous rheological behavior of suspensions of mixtures of fumed silica nanoparticles and rod-shaped cellulose nanocrystals.

### 1.1. Fumed Silica

**Figure 1:**
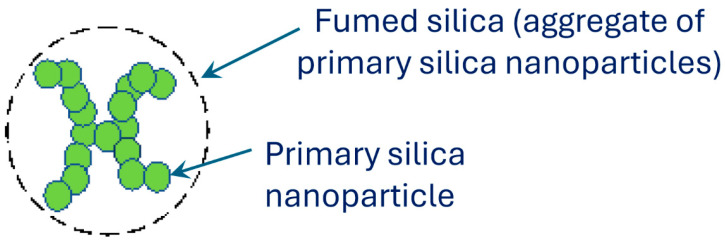
Fumed silica—an aggregate of primary silica nanoparticles.

**Figure 2:**
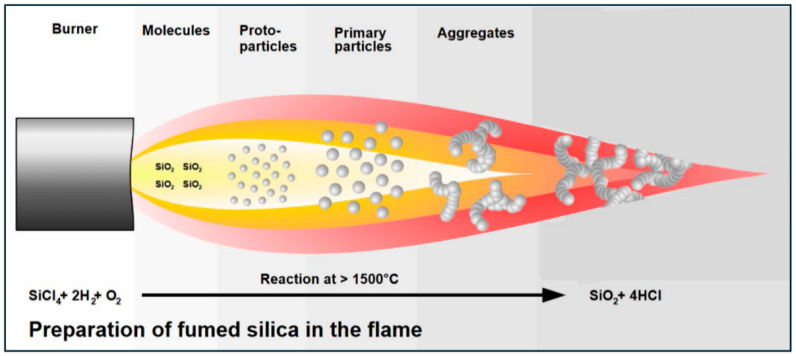
Production of fumed silica by flame hydrolysis of silicon tetrachloride.

Fumed silica is an aggregate of primary silica nanoparticles as shown schematically in Figure 1. It is a versatile nanomaterial with unique properties such as a high specific surface area. It is used extensively in industries such as cosmetics, food, and pharmaceuticals. It has excellent thickening properties. Fumed silica is produced by flame hydrolysis of silicon tetrachloride, as shown schematically in Figure 2 [8]. The silicon tetrachloride reaction when it burns in oxygen and hydrogen flames generates primary particles in molten form. The diameters of the primary particles generated are in the range of 5–50 nm [9,10,11,12]. The primary particles in molten state collide with each other and fuse together to form bigger secondary particles or aggregates. The aggregates are typically in the size range of 100 to 500 nm [9,10,11,12]. Fused silica nanoparticles acquire a negative charge when dispersed in aqueous phase as the surface of the particles are covered with silanol groups (Si-OH).

**Figure 3:**
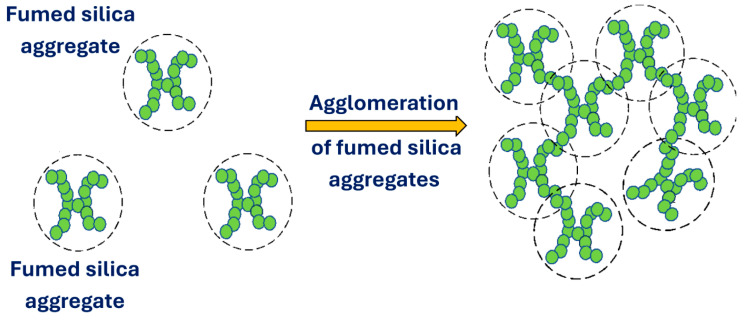
Formation of large agglomerates of fumed silica aggregates.

It should be noted that the aggregates of fumed silica often form large agglomerates when dispersed in aqueous phase at high concentrations due to the presence of attractive forces between the aggregates caused by hydrogen bonding of silanol groups. The generation of agglomerates is shown schematically in Figure 3.

### 1.2. Nanocrystalline Cellulose

Nanocrystalline cellulose (NCC) is a promising cost-effective nanomaterial with many potential applications [13,14,15,16,17,18,19]. It possesses special characteristics such as high stiffness, high tensile strength, high aspect ratio (rod-shaped), high surface area, and multiple hydroxyl groups for functionalization. Furthermore, it is non-toxic, biodegradable, and renewable. NCC can be derived from a variety of different sources of cellulose including plant waste biomass [20], tunicates [21,22], wood [23,24,25,26], cotton [27,28], algae [29,30], and bacterial cellulose [31,32]. Depending on the source of NCC and the method of production, the width of the cellulose nanocrystals can range from 3 to 50 nm, while the length of the rod-shaped nanocrystal can vary from tens of nanometers to several micrometers [33,34].

NCC is commonly produced by hydrolysis of amorphous portions of cellulose fibers by sulfuric acid. The cellulose undergoes simultaneous hydrolysis of glycosidic bonds and esterification of surface hydroxyl groups. The hydrolysis reaction breaks the cellulose chains such that the disordered regions are fully degraded and only crystalline portions remain [2]. The esterification reaction converts the surface hydroxyl groups into anionic half-ester groups. Due to the presence of anionic half-ester groups, the cellulose nanocrystals develop a negative charge when they are dispersed in water. The zeta potential of cellulose nanocrystals is generally in the range of −20 to −50 mV [35]. The high aspect ratio of rod-shaped NCC and its negative surface charge are important factors that make cellulose nanocrystals excellent modifiers of rheology. NCC thickens liquids substantially and usually imparts non-Newtonian shear-thinning characteristics to liquids [2].

## 2. Materials and Methods

### 2.1. Materials

**Figure 4:**
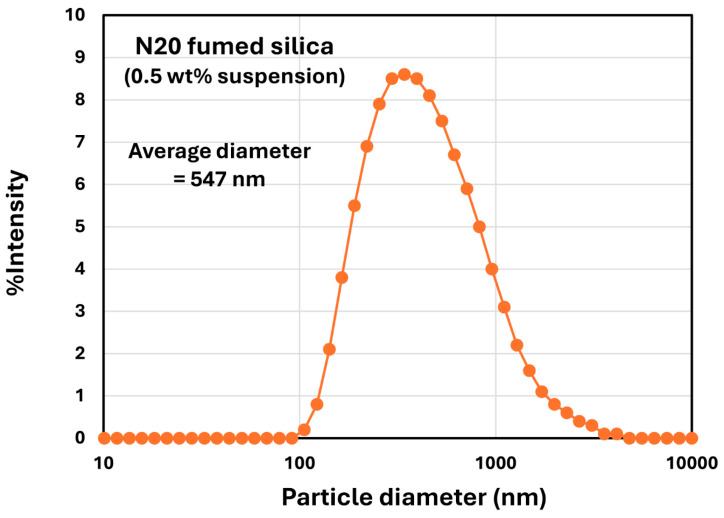
Size distribution of fumed silica aggregates obtained using dynamic light scattering.

The fumed silica was provided by Wacker Chemie AG (Munchen, Germany). The specifications for the fumed silica supplied are synthetic hydrophilic amorphous pyrogenic silica HDK N20, referred to as N20 and produced via flame hydrolysis, SiO_2_ content of 99.8 wt%, specific surface area of 175–225 m^2^/g, density of 2.2 g/cc, silanol group density of 2 SiOH/nm^2^, refractive index of 1.46, and primary particle diameter of 12 nm. Figure 4 shows the size distribution of fumed silica N20 obtained using dynamic light scattering (DLS) at the low dispersed phase concentration of 0.5 wt%. The DLS measurements were carried out using a Zetasizer Nano ZS90 instrument provided by Malvern Instruments Ltd., Worcester, UK. The average diameter of N20 aggregates was 547 nm. The zeta potential of N20 was −37.3 mV.

**Figure 5:**
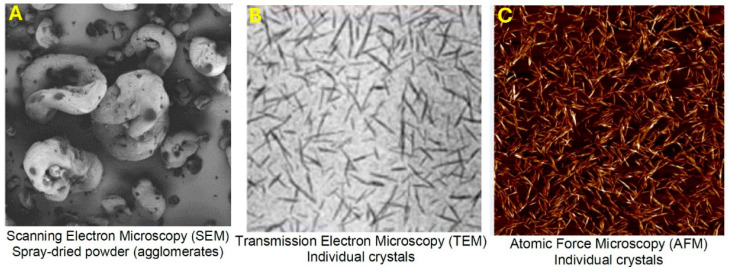
SEM, TEM, and AFM images of NCC. (**A**): SEM image of spray-dried NCC powder; (**B**): TEM image of cellulose nanocrystals; (**C**): AFM image of cellulose nanocrystals.

**Figure 6:**
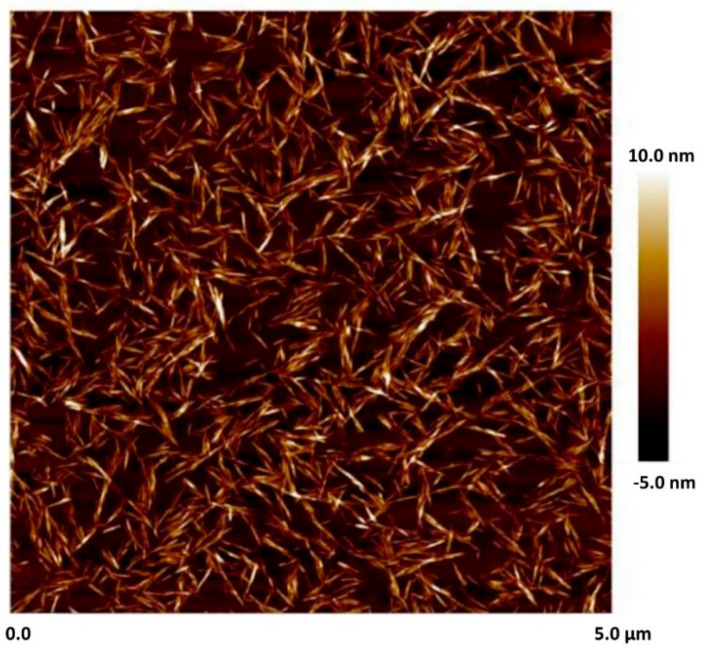
Atomic force microscopy (AFM) of nanocrystals with scale.

The nanocrystalline cellulose (NCC) was manufactured by CelluForce Inc., Windsor, ON, Canada. The trade name of NCC was NCC NCV100-NASD90. The NCC was produced using hydrolysis of wood pulp with sulfuric acid. The data sheet for NCC provided the following specifications: hydrophilic sodium-neutralized sulfated cellulose nanocrystals in spray-dried powder form, particle shape—sticks, mean length—76 nm, mean width—3.4 nm, specific surface area—500 m^2^/g, crystallinity—88%. Figure 5 shows SEM, TEM, and AFM images of NCC supplied by the company. Unfortunately, no scale was provided for the SEM and TEM images. Figure 6 shows the AFM image of NCC with scale. The nanocrystals are clearly rod- or needle-shaped particles.

### 2.2. Preparation of Suspensions of Fumed Silica (N20) and Nanocrystalline Cellulose (NCC)

The suspensions of fumed silica (N20) and nanocrystalline cellulose (NCC) were prepared in batches of about 1 kg at room temperature ( ≅23 °C). A known amount of fumed silica or NCC powder was added to a known amount of de-ionized water, and the mixture was homogenized with a variable-speed homogenizer (Gifford-Wood, model 1 L) at a fixed speed. The mixture was homogenized for about 60 min for the powder (fumed silica or nanocrystal) to disperse and mix thoroughly.

The suspensions were prepared at seven different concentrations. For the fumed silica suspensions, the concentrations investigated were 2, 3.9, 5.8, 7.7, 9.0, 9.5, and 11.3 wt%. The dispersed phase concentrations for NCC suspensions were varied as 0.99, 1.97, 2.95, 3.91, 4.86, 5.80, and 6.73 wt%.

### 2.3. Preparation of Suspensions of Mixed Fumed Silica (N20) and Nanocrystalline Cellulose (NCC)

Suspensions of mixed fumed silica N20 and nanocrystalline cellulose NCC were prepared as follows: a N20 suspension was first prepared at a fixed N20 concentration using the homogenizer. The suspension of mixed NCC and N20 was then prepared by adding a known amount of NCC to the N20 suspension while keeping the mixing of the fluid on in the homogenizer. To prepare mixed suspensions with higher NCC concentrations, the known amount of additional NCC was incorporated into an existing mixed suspension of NCC and N20. After addition of NCC, the mixing was carried out in the homogenizer at a fixed speed for about 60 min. Table 1 summarizes the concentrations of the suspensions of N20 and NCC mixtures investigated.

### 2.4. Measurement of Viscous Rheological Behavior of Suspensions

The viscous rheological behavior of suspensions of nano-additives was experimentally determined using Fann- and Haake-type viscometers with co-axial cylinder geometry. The radii of the co-axial cylinders and corresponding gap-widths are summarized in Table 2. In the Fann viscometer, the outer cylinder rotates, and the inner cylinder is kept stationary. In the Haake viscometer, the outer cylinder is kept stationary, and the inner cylinder rotates. The Fann viscometer has 12 speeds covering the range of 0.9 to 600 rpm. The Haake viscometer has 30 speeds covering the range of 0.01 to 512 rpm. The calibrations of the viscometers were carried out using viscosity standards of known viscosities. The shear rate range and calibration relations of the viscometers are given in Table 3. The rheological measurements of suspensions were performed at room temperature.

## 3. Results and Discussion

### 3.1. Rheology of Suspensions of Pure Fumed Silica and Pure Nanocrystalline Cellulose

**Figure 7:**
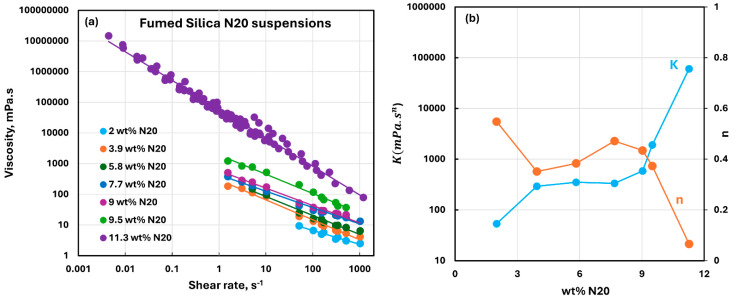
Viscous rheological behavior of suspensions of pure fumed silica N20. (**a**) viscosity versus shear rate plots on log-log scale. (**b**) power-law parameters, consistency index K and flow behavior index n.

The viscous rheological behavior of suspensions of pure fumed silica N20 is shown in Figure 7. The fumed silica suspensions are non-Newtonian shear-thinning, that is, the viscosity decreases with the increase in shear rate at any given N20 concentration. The viscosity versus shear rate plots are linear, indicating that the suspensions follow the power-law model given as [36]:

(1) $$\eta =\tau /\dot{\gamma }=K{\dot{\gamma }}^{n-1}$$

where η is viscosity, τ is shear stress, $\dot{\gamma }$ is shear rate, K is consistency index, and n is flow behavior index. The consistency index K is a measure of the consistency of fluid and flow behavior index n is a measure of the flow behavior of fluid. The flow behavior index is unity for Newtonian fluids. For non-Newtonian fluids, the flow behavior index is different from unity. For shear-thinning fluids, n<1 and for shear-thickening fluids, n>1. Note that the viscosity versus shear rate plots shift upwards (see Figure 7a) with the increase in fumed silica concentration indicating an increase in viscosity with the increase in N20 concentration. The power-law constants of suspensions of fumed silica are plotted in Figure 7b. The consistency index initially rises sharply at low N20 concentrations, then levels off at intermediate N20 concentrations, and rises sharply again at high N20 concentrations. The flow behavior index n is less than 1 for all suspensions, indicating the shear-thinning nature of suspensions. The flow behavior index drops initially at low N20 concentrations, then increases slightly at intermediate N20 concentrations, and drops sharply again at high N20 concentrations. Thus, the suspensions of fumed silica become highly viscous and severely shear-thinning at high N20 concentrations.

**Figure 8:**
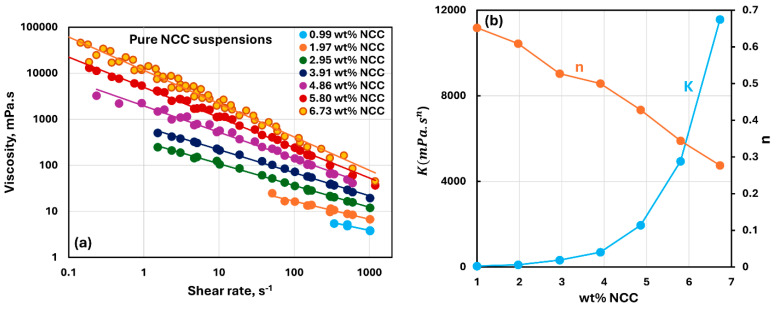
Viscous rheological behavior of suspensions of pure nanocrystalline cellulose NCC. (**a**) viscosity versus shear rate plots on log-log scale. (**b**) power-law parameters, consistency index K and flow behavior index n.

The viscous rheological behavior of suspensions of pure nanocrystalline cellulose NCC is shown in Figure 8. Like fumed silica suspensions, the NCC suspensions are non-Newtonian shear-thinning, that is, at any given NCC concentration, the viscosity decreases as the shear rate is increased. Furthermore, the viscosity versus shear rate plots are linear, indicating that the suspensions obey the power-law model (Equation (1)). The consistency index increases sharply with the NCC concentration, especially when NCC concentration is larger than 4 wt%. The flow behavior index decreases almost linearly with the increase in NCC concentration indicating that the degree of shear-thinning increases linearly with the increase in NCC concentration. This is consistent with previous studies on the rheology of NCC suspensions [37].

**Figure 9:**
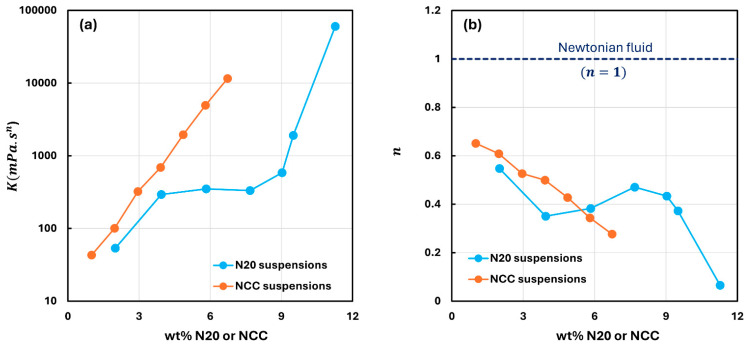
Comparison of power-law parameters of pure fumed silica and pure nanocrystalline cellulose suspensions. (**a**) consistency index K. (**b**) flow behavior index n.

Figure 9 compares the rheological power-law parameters of suspensions of pure fumed silica and pure nanocrystalline cellulose. The NCC suspensions have a much higher consistency, that is, they are more viscous when comparison is made at the same wt% concentration of additive. This is especially true at high concentrations of additives. The degree of shear-thinning in N20 and NCC suspensions is similar up to additive concentrations of about 6 wt%. At higher concentrations, NCC suspensions appear to be more shear-thinning and have lower values of flow behavior index as compared with fumed silica suspensions at the same concentration of dispersed phase. It is important to note that the mechanisms of shear-thinning in fumed silica and NCC suspensions are different. The shear-thinning in fumed silica suspensions is due to the break-up of large agglomerates of fumed silica aggregates with the increase in shear rate, whereas shear-thinning in NCC suspensions is likely caused by the orientation of needle-shaped nanocrystals in the flow direction upon the increase in shear rate.

### 3.2. Rheology of Suspensions of Mixtures of Fumed Silica and Nanocrystalline Cellulose

**Figure 10:**
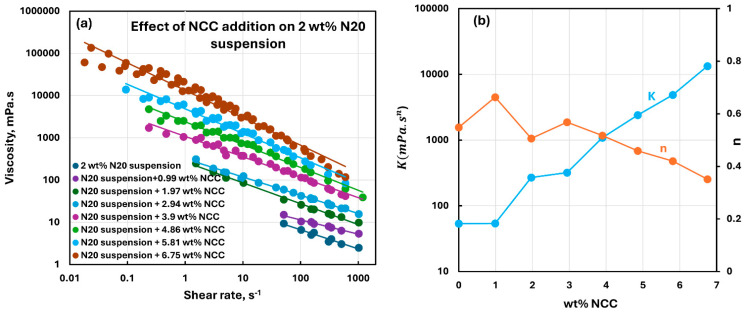
Viscous rheological behavior of suspensions of mixed fumed silica N20 and nanocrystalline cellulose NCC at fixed N20 concentration of 2 wt%. (**a**) viscosity versus shear rate plots on log-log scale. (**b**) power-law parameters, consistency index K and flow behavior index n.

**Figure 11:**
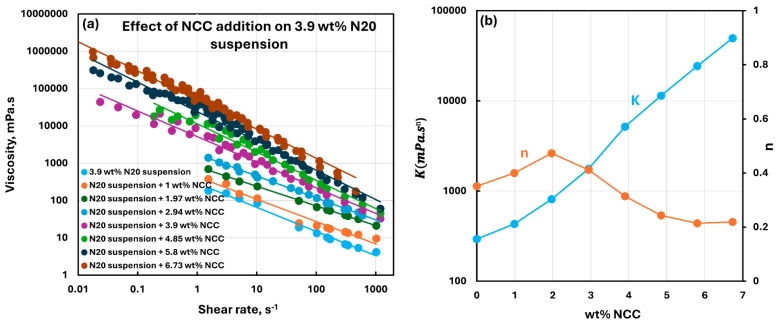
Viscous rheological behavior of suspensions of mixed fumed silica N20 and nanocrystalline cellulose NCC at fixed N20 concentration of 3.9 wt%. (**a**) viscosity versus shear rate plots on log-log scale. (**b**) power-law parameters, consistency index K and flow behavior index n.

**Figure 12:**
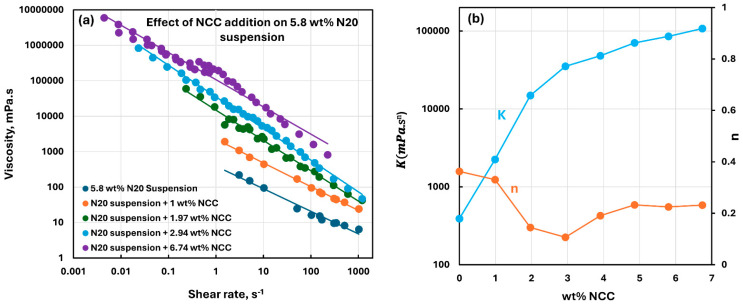
Viscous rheological behavior of suspensions of mixed fumed silica N20 and nanocrystalline cellulose NCC at fixed N20 concentration of 5.8 wt%. (**a**) viscosity versus shear rate plots on log-log scale. (**b**) power-law parameters, consistency index K and flow behavior index n.

**Figure 13:**
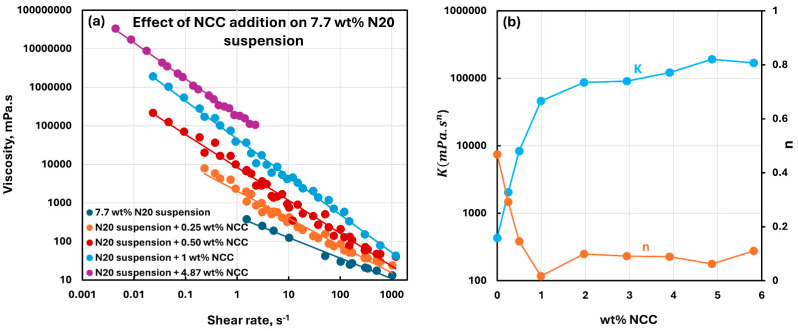
Viscous rheological behavior of suspensions of mixed fumed silica N20 and nanocrystalline cellulose NCC at fixed N20 concentration of 7.7 wt%. (**a**) viscosity versus shear rate plots on log-log scale. (**b**) power-law parameters, consistency index K and flow behavior index n.

**Figure 14:**
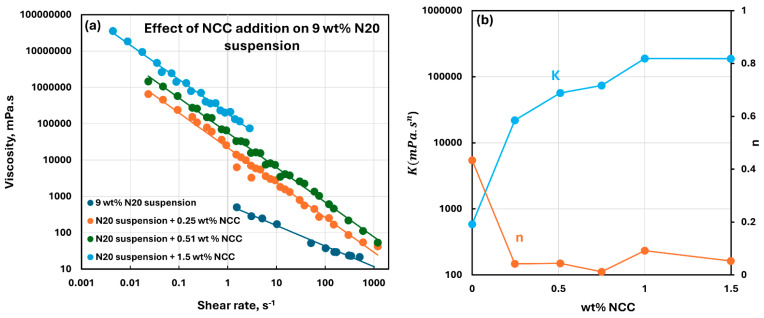
Viscous rheological behavior of suspensions of mixed fumed silica N20 and nanocrystalline cellulose NCC at fixed N20 concentration of 9 wt%. (**a**) viscosity versus shear rate plots on log-log scale. (**b**) power-law parameters, consistency index K and flow behavior index n.

Figure 10, Figure 11, Figure 12, Figure 13 and Figure 14 show the viscous rheological behavior of suspensions of mixed nano-additives, that is, fumed silica and NCC. In any given figure, the fumed silica concentration is fixed and the NCC concentration is varied. Like the suspensions of pure additives, the suspensions of mixed additives are also non-Newtonian shear-thinning. Furthermore, all suspensions of mixed additives follow the power-law model, that is, a linear relationship between viscosity and shear rate on a log-log scale. At any given N20 concentration, the consistency index rises sharply, and the flow behavior index falls substantially with the incorporation of NCC into N20 suspension.

**Figure 15:**
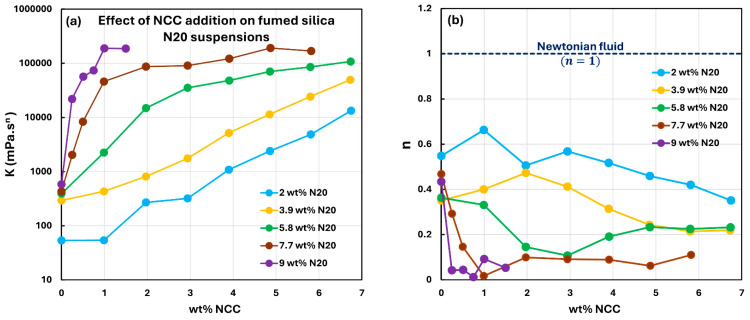
Comparison of the rheological power-law parameters, consistency index K and flow behavior index n, for suspensions of mixed additives (N20 and NCC) at different concentrations of fumed silica N20. (**a**) consistency index K. (**b**) flow behavior index n.

Figure 15 compares the rheological power-law parameters, consistency index K and flow behavior index n, for suspensions of mixed additives at different concentrations of fumed silica N20. At low N20 concentrations ( ≤4 wt%), the consistency index rises with the addition of NCC concentration almost linearly on a semi-log scale. At higher N20 concentrations, the consistency index initially increases sharply with the increase in NCC concentration but tends to level off at high NCC concentrations. At any given NCC concentration, the consistency index increases with the increase in N20 concentration. Thus, both N20 and NCC concentrations strongly affect the consistency index. The flow behavior index decreases with the increases in both N20 and NCC concentrations.

**Figure 16:**
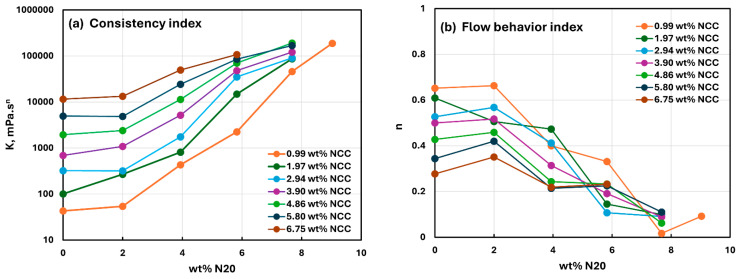
Comparison of the rheological power-law parameters, consistency index K and flow behavior index n, for suspensions of mixed additives (N20 and NCC) at different concentrations of nanocrystalline cellulose NCC. (**a**) consistency index K. (**b**) flow behavior index n.

Figure 16 compares the viscous rheological properties of suspensions of mixed additives (N20 and NCC) on a different basis. Now the power-law parameters are plotted as functions of N20 concentration at fixed NCC concentrations. The consistency index increases with the increase in N20 concentration at a fixed NCC concentration. With the increase in NCC concentration, the consistency index versus N20 concentration plot shifts upwards toward higher consistency values. The flow behavior index decreases with the increase in N20 concentration at a fixed NCC concentration. With the increase in NCC concentration, the flow behavior index versus N20 concentration plot generally shifts downwards toward lower n values indicating an increase in the shear-thinning of suspension.

### 3.3. Visual Inspection of Suspensions

**Figure 17:**
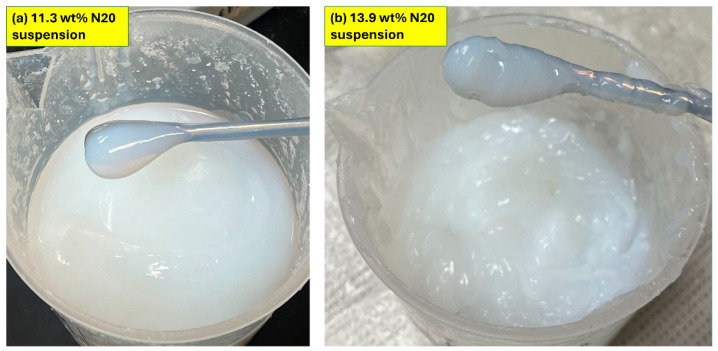
Fumed silica N20 suspensions at different N20 concentrations.

**Figure 18:**
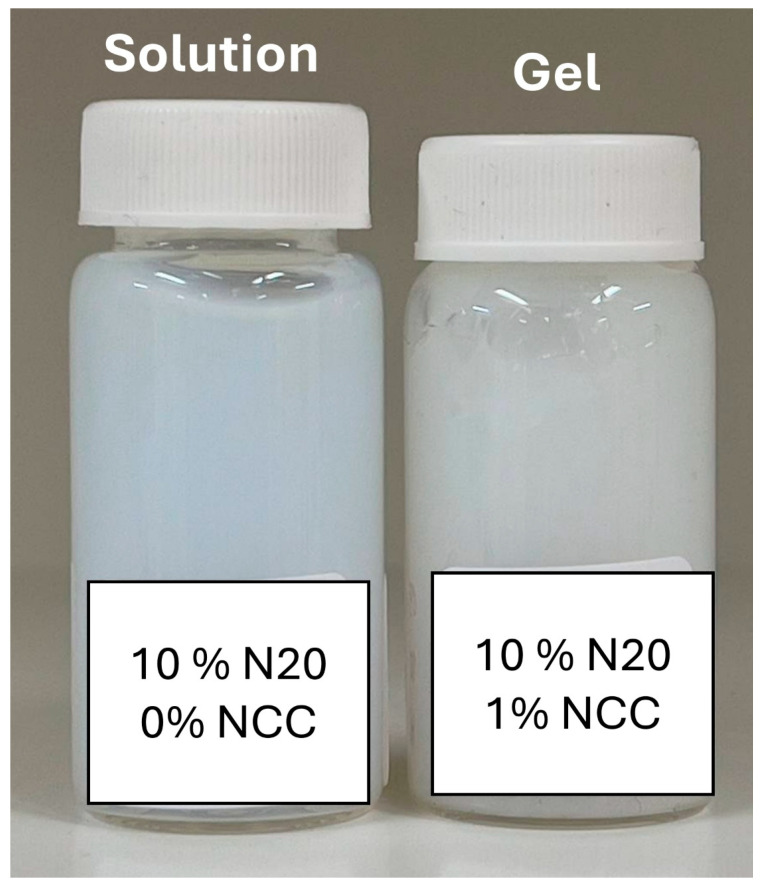
Change in consistency of fumed silica suspension upon addition of nanocrystalline cellulose.

Figure 17 shows samples of fumed silica N20 suspensions at two N20 concentrations. The fumed silica suspensions were fluid at N20 concentrations of less than about 11.5 wt%. At higher N20 concentrations, the fumed silica suspensions were gels with a paste-like consistency. Upon addition of nanocrystalline cellulose to fumed silica suspensions (see Figure 18), the consistency of the fluid-like N20 suspension changes to gel-like material at high concentrations of N20 and NCC.

### 3.4. Reliability and Error Analysis of Rheological Measurements

It is important for the rheological measurements of suspensions to be reliable and accurate that the inertial effects and wall effects (also called slip effects) are absent, and there is negligible sedimentation of particles. The inertial effects are negligible when the particle Reynolds number (${Re}_{p}$) is very small, that is, ${Re}_{p}\ll 1$. For wall effects to be negligible, the ratio of particle diameter ($d_{p}$) to gap-width (w) where fluid shearing takes place should be small, that is, $d_{p}/w<0.1$. Sedimentation of suspension particles can be neglected if the settling velocity of particles under the influence of gravity is very small.

The particle Reynolds numbers for fumed silica and nanocrystals were calculated as follows:

(2) $${Re}_{p}=\frac{\rho_{c}\dot{\gamma }r_{p}^{2}}{\eta_{c}}$$

where $\rho_{c}$ is density, $\eta_{c}$ is viscosity of continuous phase fluid (matrix fluid), $\dot{\gamma }$ is shear rate, and $r_{p}$ is particle radius. The maximum shear rate in our experiments was about $\dot{\gamma }=10^{3}$ s^−1^. For fumed silica, rp = 273.5 nm. Hence, for fumed silica:

(3) $${Re}_{p}=\frac{\rho_{c}\dot{\gamma }r_{p}^{2}}{\eta_{c}}=\frac{10^{3}\times 10^{3}\times {\left(273.5\times 10^{-9}\right)}^{2}}{10^{-3}}=7.48\times 10^{-5}\;$$

Note that $\rho_{c}$ and $\eta_{c}$ are approximately 10^3^ kg/m^3^ and 10^−3^ Pa·s for water as our continuous phase. For the rod-shaped NCC, we take the maximum dimension (length) as $r_{p}$ in the calculation of the particle Reynolds number, that is, $r_{p}=76$ nm for the NCC used. Hence, for NCC:

(4) $${Re}_{p}=\frac{\rho_{c}\dot{\gamma }r_{p}^{2}}{\eta_{c}}=\frac{10^{3}\times 10^{3}\times {\left(76\times 10^{-9}\right)}^{2}}{10^{-3}}=5.78\times 10^{-6}$$

Thus, the flow in our experiments could be characterized as an extremely creeping flow with negligible inertial effects, as ${Re}_{p}\ll 1$.

To determine the possibility of wall effects, the ratio of particle diameter to gap-width was calculated. The minimum gap-width in our measurements was 0.10 cm. Thus, for fumed silica:

(5) $$ratio=\frac{d_{p}}{w}=\frac{547\;nm}{0.10\;cm}=5.47\times 10^{-4}$$

This ratio is too small for wall effects to become relevant. For NCC, the ratio was even smaller than the value calculated above, as NCC dimensions were smaller than that of fumed silica.

The settling velocity of particles was estimated using Stokes’ law, which is applicable under creeping flow conditions [7]:

(6) $$U_{t}=\frac{\left(\rho_{p}-\rho_{c}\right)gd_{p}^{2}}{18\eta_{c}}$$

where $U_{t}$ is the settling velocity of a particle, $\rho_{p}$ is the density of particle, and g is acceleration due to gravity. For fumed silica, $U_{t}$ is estimated to be:

(7) $$U_{t}=\frac{\left(2.2-1\right)\left(10^{3}\right)\left(9.8\right){\left(547\times 10^{-9}\right)}^{2}}{18\times 10^{-3}}=1.95\times 10^{-7}\;m/s$$

For NCC, $U_{t}$ is estimated to be:

(8) $$U_{t}=\frac{\left(1.5-1\right)\left(10^{3}\right)\left(9.8\right){\left(76\times 10^{-9}\right)}^{2}}{18\times 10^{-3}}=1.57\times 10^{-9}\;m/s$$

The sedimentation velocities of particles are too small for sedimentation to be relevant during rheological measurements.

**Figure 19:**
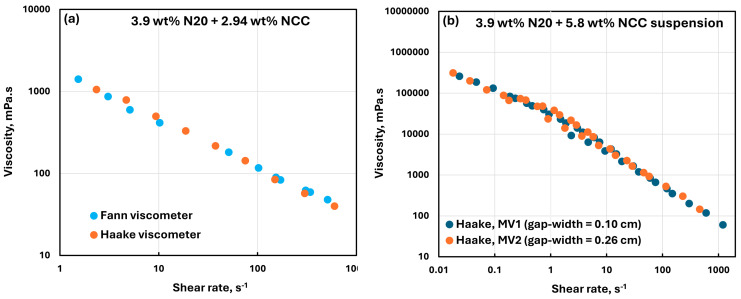
Comparison of rheological data obtained from different viscometers and different gap-widths for the same fluid (3.9 wt% N20 with 2.94 wt% NCC). (**a**) comparison with different viscometers. (**b**) comparison with different gap-widths.

The reliability of rheological measurements was confirmed by comparing the rheological data obtained from two different viscometers (Fann and Haake) for the same fluid. For example, Figure 19a compares the viscosity data obtained from the Fann and Haake viscometers for 3.9 wt% N20 suspension containing 2.94 wt% NCC. The data obtained from the different viscometers overlap with each other, indicating that the measurements are reliable. Note that in the Fann viscometer, the outer cylinder rotates and the inner cylinder (bob) is held stationary. In the Haake viscometer, it is the other way round, that is, the outer cylinder is stationary and the inner cylinder rotates. Figure 19b shows the data for the same fluid (3.9 wt% N20 suspension containing 5.8 wt% NCC) using the Haake viscometer with different gap-widths. The gap-width between the inner cylinder and outer cylinder of the viscometer is 0.10 cm for the MV1 system and 0.26 cm for the MV2 system. Even though the gap-widths are very different, the rheological data overlap, indicating that there were no wall or slip effects encountered in the measurements.

The goodness-of-fit of the power-law model, Equation (1), was evaluated by the calculation of coefficient of determination $R^{2}$. As an example, Table 4 shows the power-law constants, K and n, and the corresponding values of $R^{2}$ for 2 wt% N20 system containing different concentrations of added NCC. The $R^{2}$ values are generally very high, confirming that the power-law model is an appropriate model to describe the rheological data. At the highest NCC concentration of 6.75 wt%, however, $R^{2}$ value is the lowest, that is, 0.7631. Thus, the power-law model describes the rheological data at the highest NCC concentration of 6.75 wt% only approximately.

Table 5 summarizes the errors between power-law model predictions and measured values for 2 wt% N20 system containing different concentrations of NCC. The values of root mean square error (RMSE), average percent error (APRE), and absolute average percent error (AAPRE) are given at each concentration of NCC. RMSE, APRE, and AARE are defined as follows:

(9) $$\;RMSE=\frac{1}{N}\sum_{i=1}^{i=N}{\left[{\left(\eta_{model}\right)}_{i}-{\left(\eta_{expt}\right)}_{i}\right]}^{2}\;$$

(10) $$APRE=\frac{1}{N}\sum_{i=1}^{i=N}\left[\frac{{\left(\eta_{model}\right)}_{i}-{\left(\eta_{expt}\right)}_{i}}{{\left(\eta_{expt}\right)}_{i}}\right]\times 100$$

(11) $$AAPRE=\frac{1}{N}\sum_{i=1}^{i=N}abs\left[\frac{{\left(\eta_{model}\right)}_{i}-{\left(\eta_{expt}\right)}_{i}}{{\left(\eta_{expt}\right)}_{i}}\right]\times 100$$

where $\eta_{model}$ is the viscosity predicted from the power-law model and $\eta_{expt}$ is the corresponding measured viscosity value through experiments. The APRE is less than 2% and AAPRE is less than 15% for NCC concentrations of less than 6 wt%. At the highest NCC concentration of 6.75 wt% in a 2 wt% N20 system, the errors are somewhat large. Thus, the power-law model is only an approximation at 6.75 wt% NCC in a 2 wt% N20 system. Note that the R^2^ value is also the lowest at this NCC concentration (see Table 4). The error analysis presented here is applicable to all suspensions of mixed NCC and N20 additives investigated in this work.

## 4. Conclusions

The viscous rheological behavior of suspensions of pure fumed silica N20, pure nanocrystalline cellulose NCC, and mixtures of fumed silica N20 and nanocrystalline cellulose NCC were studied experimentally. The following conclusions can be drawn based on this study:

• Suspensions of fumed silica are non-Newtonian shear-thinning. They obey the power-law model over the N20 concentration range of 2 to 11.3 wt%. With the increase in N20 concentration, both the consistency and the degree of shear-thinning of suspension are enhanced.
• The suspensions of cellulose nanocrystals are also non-Newtonian shear-thinning, and they follow the power-law model over the NCC concentration range of 0.99 to 6.73 wt%. With the increase in NCC concentration, both the consistency and the degree of shear-thinning of suspension are enhanced.
• The suspensions of mixed additives, that is, N20 and NCC, are non-Newtonian shear-thinning. The power-law model describes the rheological behavior of the mixed suspension systems well in most cases. In some cases, especially at high concentrations of NCC in the mixed N20 and NCC suspensions, the power-law model describes the rheological data only approximately. The consistency and level of shear-thinning in suspensions of mixed additives are strongly dependent on the concentrations of both additives. The consistency and the level of shear-thinning increase substantially with the increases in N20 and NCC concentrations.
• The mechanisms of shear-thinning in suspensions are reasoned to be as follows: With the increase in shear rate, the rod-shaped cellulose nanocrystals become aligned in the direction of flow and hence offer less resistance to flow. Furthermore, the large agglomerates of fumed silica aggregates undergo breakup with the increase in shear rate, resulting in a reduction in viscosity.
• In future work, dynamic rheology of suspensions of mixed nanoparticle/nanocrystal additives will be explored.

## Figures and Tables

**Table 1:** Concentrations of suspensions of N20 and NCC mixtures investigated.

Fumed Silica (N20) Concentration in N20–Water Suspension (wt%)	Concentration of Cellulose Nanocrystals (NCC) in Mixed N20–NCC–Water Suspension (wt%)
2.0	Seven concentrations: 0.99, 1.97, 2.94, 3.9, 4.86, 5.81, 6.75
3.9	Seven concentrations: 0.99, 1.97, 2.94, 3.9, 4.85, 5.8, 6.73
5.8	Seven concentrations: 0.99, 1.97, 2.94, 3.91, 4.86, 5.80, 6.74
7.7	Eight concentrations: 0.25, 0.50, 0.99, 1.97, 2.94, 3.91, 4.87, 5.82
9.0	Five concentrations: 0.247, 0.51, 0.75, 1.0, 1.498
9.5	No NCC added
11.3	No NCC added

**Table 2:** Radii and gap-widths of co-axial cylinders of viscometers.

Viscometer	$\mathrm{Radius}\;\mathrm{of}\;\mathrm{Inner}\;\mathrm{Cylinder},\;R_{i}$ (cm)	Radius of Outer Cylinder , R o ( c m )	Length of Inner Cylinder (cm)	Gap-Width Between Cylinders (cm)
Fann viscometer	1.72	1.84	3.8	0.12
Haake viscometer with MV I bob	2.00	2.1	6.0	0.10
Haake viscometer with MV II bob	1.84	2.1	6.0	0.26
Haake viscometer with MV III bob	1.52	2.1	6.0	0.58

**Table 3:** Shear rate range and calibrations of the viscometers.

Viscometer	Shear Rate, s^−1^	Shear Rate Range of Device, s^−1^	Shear Stress, mPa
Fann viscometer with R1 bob	1.7023 × r p m	1.53 – 1021.38	τ = 98.955 D R − 212.82
Haake viscometer with MV I bob	2.34 × r p m	0.0234 – 1198.08	τ = 898.07 D R + 133.38
Haake viscometer with MV II bob	0.90 × r p m	$9\times 10^{-3}–460.8$	τ = 1223.3 D R − 611.85
Haake viscometer with MV III bob	0.44 × r p m	$4.4\times 10^{-3}–225.28$	τ = 2352.9 D R − 1115.8

DR refers to dial reading

**Table 4:** Power-law constants and R^2^ values for 2 wt% N20 system with different concentrations of NCC.

NCC Concentration (wt%)	K , m P a s n	n	R 2
0	53.48	0.548	0.9769
0.99	54	0.663	0.9738
1.97	269.24	0.506	0.9911
2.94	319.76	0.568	0.9791
3.90	1083.5	0.517	0.9582
4.86	2400	0.459	0.9488
5.81	4859.2	0.42	0.945
6.75	13,331	0.351	0.7631

**Table 5:** Errors between power-law model predictions and measured values for 2 wt% N20 system with different concentrations of NCC.

NCC Concentration (wt%)	RMSE (mPa.s)	APRE (%)	A A P R E ( % )
0	0.33	0.38	6.43
0.99	0.48	0.22	3.86
1.97	9.17	0.34	5.87
2.94	11.55	0.12	3.96
3.90	114.97	0.94	9.87
4.86	345.22	1.20	12.59
5.81	1354.89	1.74	15.34
6.75	19,259.76	6.08	27.14

## Data Availability

The raw data supporting the conclusions of this article will be made available by the authors on request.
